# A Novel Method for Quantifying the Inhaled Dose of Air Pollutants Based on Heart Rate, Breathing Rate and Forced Vital Capacity

**DOI:** 10.1371/journal.pone.0147578

**Published:** 2016-01-25

**Authors:** Roby Greenwald, Matthew J. Hayat, Jerusha Barton, Anastasia Lopukhin

**Affiliations:** 1 Division of Environmental Health, School of Public Health, Georgia State University, Atlanta, Georgia, United States of America; 2 Department of Environmental Health, Rollins School of Public Health, Emory University, Atlanta, Georgia, United States of America; 3 Division of Epidemiology and Biostatistics, School of Public Health, Georgia State University, Atlanta, Georgia, United States of America; Hasselt University, BELGIUM

## Abstract

To better understand the interaction of physical activity and air pollution exposure, it is important to quantify the change in ventilation rate incurred by activity. In this paper, we describe a method for estimating ventilation using easily-measured variables such as heart rate (HR), breathing rate (f_B_), and forced vital capacity (FVC). We recruited healthy adolescents to use a treadmill while we continuously measured HR, f_B_, and the tidal volume (V_T_) of each breath. Participants began at rest then walked and ran at increasing speed until HR was 160–180 beats per minute followed by a cool down period. The novel feature of this method is that minute ventilation (${\dot{V}}_{E}$) was normalized by FVC. We used general linear mixed models with a random effect for subject and identified nine potential predictor variables that influence either ${\dot{V}}_{E}$ or FVC. We assessed predictive performance with a five-fold cross-validation procedure. We used a brute force selection process to identify the best performing models based on cross-validation percent error, the Akaike Information Criterion and the p-value of parameter estimates. We found a two-predictor model including HR and f_B_ to have the best predictive performance (${\dot{V}}_{E}$/FVC = -4.247+0.0595HR+0.226f_B_, mean percent error = 8.1±29%); however, given the ubiquity of HR measurements, a one-predictor model including HR may also be useful (${\dot{V}}_{E}$/FVC = -3.859+0.101HR, mean percent error = 11.3±36%).

## Introduction

The negative health consequences related to air pollution exposure are widely acknowledged and include cardiovascular, respiratory, and nervous system health effects [1–6]. Similarly, obesity is associated with many negative health outcomes including cardiovascular disease, type 2 diabetes, several different types of cancer, and many co-morbidities [7]. Physical activity is an important element in reducing the mortality and morbidity risks of obesity and improving overall well-being[8–12]. Physical activity increases the ventilation rate and in the presence of air pollution also increases the inhaled dose of air pollution. This issue is of particular concern in the field of active transportation (i.e., walking or bicycling), but also has implications for occupational or recreational activity in places of poor air quality. In order to better understand the interaction of physical activity with air pollution exposure, it is important to improve existing methods of quantifying the change in inhaled dose related to physical activity.

Numerous previous studies have examined the topic of ventilation rate and physical activity in the context of air pollution exposure. An early investigation by Samet et al. [13,14] used heart rate (HR) to predict minute ventilation (the rate of expired ventilation, ${\dot{V}}_{E}$) in a panel of 58 adults and children in New Mexico who were engaged in three types of activities. They found that though the regression of ${\dot{V}}_{E}$ to HR produced approximately parallel curves for all subjects, there was as much as a 2–3 fold difference in ${\dot{V}}_{E}$ at the same HR between subjects. Zuurbier et al. [15] performed the same measurements on a panel of commuters in the Netherlands in order to estimate differences in exposure between commute types and had very similar findings. Bernmark et al. [16] performed these measurements on a panel of five bicycle messengers in Stockholm and also found large inter-subject variability. Each of these studies demonstrated the need to regress ${\dot{V}}_{E}$ to HR for each subject independently in order to obtain an equation that could then be used to estimate the inhaled volume of air in an ambulatory setting. Van Wijnen et al. [17] used a different approach to assess inhaled dose in a panel of cyclists, drivers and pedestrians in Amsterdam: they directly measured ventilation by having subjects exhale through a face mask connected to a gas meter. This study also found a variety of ventilation rates, but the mask employed to obtain these measurements is not conducive to large-scale field studies of persons in natural settings. Beals et al. [18] examined ${\dot{V}}_{E}$ in 160 children and adults in California with a goal of quantifying the variability of ${\dot{V}}_{E}$ across the population. They determined the distribution of ventilation rates for different types of activities and concluded there was a large amount of inter-subject variability in ventilation rates based on factors such as age, sex, and the amount of physical activity.

Two recently published studies have examined the air pollution inhaled dose in various settings using similar methodology: performing measurements of ${\dot{V}}_{E}$ and HR in a controlled setting, developing a regression model for each subject, then using ambulatory HR measurements to estimate ${\dot{V}}_{E}$ and inhaled dose in a natural setting. Ramos et al. [19] estimated ${\dot{V}}_{E}$ and inhaled dose in 20 volunteers participating in indoor physical activities by performing the ${\dot{V}}_{E}$ and HR regression for each subject individually. Cozza et al. [20] estimated ${\dot{V}}_{E}$ and inhaled dose in an occupational setting for two groups of participants, one group in a city street setting and the other in a forest park setting. In this study, a linear mixed effects model with random intercepts and slopes was developed for each group and again demonstrated the limitations of inter-subject variability in the relationship of ${\dot{V}}_{E}$ and HR.

A third recent study by Faria et al. [21] used a unique approach to estimate ${\dot{V}}_{E}$ and inhaled dose in pedestrians with a regression model based on power expenditure. This model requires information pertaining to pedestrian velocity and the incline gradient. Both of these predictors must be measured using dedicated equipment. It was validated in three subjects with a percent difference between predictions and observations ranging from 3–34%.

In light of this previous work, the purpose of this study was to develop a simple method to easily quantify ${\dot{V}}_{E}$ based on data that is readily-available or easy to measure in the field using inexpensive equipment. An important goal was to eliminate the need to perform a regression of ${\dot{V}}_{E}$ and HR for each subject in a laboratory setting. The method we describe here predicts ${\dot{V}}_{E}$ using widely-measured HR (in addition to numerous medical devices, there are many consumer-grade wearable devices that measure and record HR data) and the less ubiquitous though still easily-measured breathing rate (or breath frequency, f_B_). The key novel feature of this method is that ${\dot{V}}_{E}$ is normalized to forced vital capacity (FVC), an easily measured or estimated value closely related to an individual’s functional lung volume. Since FVC is largely determined by a person’s age, height, sex, and race, this normalization procedure facilitates comparison of data from persons of various body morphologies and lung sizes.

## Methods

### Study Design

This study was approved by the Emory University Institutional Review Board. Written consent was obtained from all participants. For participants under 18 years of age, this consisted of written parental consent along with written assent by the subject. Subjects 18 years or older provided their own written consent. A repeated measures study design was used. This study was nested within a larger ongoing project examining air pollution exposure in adolescent athletes, and therefore, in order to match the population of the parent study, we recruited a panel of 15 healthy adolescents who participate in extracurricular athletic activities from a high school in Atlanta, Georgia.

### Data collection

Study participants engaged in physical activity in the form of walking and running on a treadmill in the high school’s indoor sports facility. A treadmill test was selected over a stationary cycling test in the interest of similarity to the types of activities performed in the main study. The treadmill test consisted of about a minute at rest, a minute of walking, and 4–5 minutes of running at gradually increasing speed until HR reached 160–180 beats per minute. At this point, subjects decreased to a jog and then a walk for a cool down period of 3–4 minutes.

During the entire treadmill test, subjects wore a chest strap with an integrated electronic device for the continuous measurement of HR and f_B_ as well as an accelerometer for measuring motion (BioHarness 3, Zephyr Technology Corp.). This device measures the electrocardiogram through two leads, and f_B_ is measured by a chest expansion sensor, both embedded in the strap. The BioHarness is equipped with a Bluetooth® transmitter, and HR and f_B_ were monitored in real-time during the treadmill test by study personnel using a laptop computer.

We measured the tidal volume (V_T_) of each exhalation throughout the entire treadmill test by connecting the exhalation port on a face mask (Training Mask 2.0, Training Mask) to a respirometer (Wright Mark 8, nSpire Health Inc.). This model of mask fits tightly against the face and is designed to be worn during intense physical activity. We saw no evidence of air flow leakage around the mask even at high inspiratory or expiratory flow. We removed the resistance valves that are used with this mask for training purposes, and during pre-test evaluation, we detected little resistance to either inspiratory or expiratory flow using the mask and respirometer set-up, even at high ventilation rate. Subjects were instructed to stop the test and remove the mask immediately if they experienced difficulty breathing, though this did not happen in practice.

Both before and after the treadmill test, subjects performed three spirometry maneuvers using a handheld spirometer (EasyOne Plus, ndd Medical Technologies Inc.) under the direction of trained study staff following the recommended procedures of the American Thoracic Society. The forced vital capacity maneuver (FVC) was used, and measurements used for data analysis were FVC, forced expiratory volume in one second (FEV_1_), and the FEV_1_/FVC ratio. For all analyses, we used the data from the pre-test maneuver with the highest FVC and an acceptable flow-volume loop.

### Data processing

The BioHarness proprietary software creates a summary file containing data with 1-second time resolution, including HR (beats per minute), f_B_ (breaths per minute) and an activity parameter (ACT) equal to the vector addition of three-dimensional acceleration expressed as a fraction of standard gravity. We then examined the respirometer data, and each time an exhalation was observed, the total volume of that breath (V_T_) was added to the summary file at the time the exhalation began. This resulted in a new data file with 1-second time resolution containing HR, f_B_, ACT and V_T_ data. From this compilation, we created four time-averaged data sets: 60-, 30-, and 15-second averages, and an “individual breath” data set of averages since the previous breath (typically 1–7 seconds). For all data sets, each row contained the corresponding average of HR, f_B_, and ACT as well as V_T_ data. We then added several additional columns of calculated values to the data sets, namely ${\dot{V}}_{E}$, both ${\dot{V}}_{E}$ and V_T_ normalized to FVC, and a dichotomous indicator variable labeled “warm” (for warm up or cool down) equal to zero if HR was increasing and one if decreasing. Due to the design of the treadmill test, in most cases, HR monotonically increased to the maximum value and then decreased during the cool down period. It is important to point out that ${\dot{V}}_{E}$ in these data sets is calculated as V_T_ times f_B_ and therefore represents the mean volumetric flow rate during the averaging period rather than the literal volume of air inhaled in one minute.

### Statistical methods

We used general linear mixed models to model the multiple ${\dot{V}}_{E}$/FVC measurements taken on each participant. This is an appropriate statistical model to use with repeated measures data [22]. We included a random effect for subject to account for the multiple measurements taken over time on each subject. To explore the sensitivity of model estimates to within-subject covariance structure, we ran all models using variance components, compound symmetry, unstructured, and first order heterogeneous autoregressive covariance structures. One subject in particular appeared to exhibit increased variance in ${\dot{V}}_{E}$ at higher values, and consequently the unstructured covariance matrix was unable to produce parameter estimates when this subject was included. Otherwise, parameter estimates were not found to be sensitive to covariance structure. We used the PROC MIXED procedure of SAS v9.3 (SAS Institute Inc, Cary, NC) and the lme4 package for R v3.2.0 (R Foundation for Statistical Computing) to take advantage of both package’s respective features. Specifically, R was used to construct programming loops to evaluate hundreds of models in a single pass during the model selection process while SAS was used for the evaluation of covariance structures and calculation of p values for parameter estimates. All presented model results are from SAS using the variance components structure. The level of significance was set *a priori* at 0.05.

We employed a brute force method of model selection wherein all possible combinations of predictors were evaluated. We included in the full model all measurements that could plausibly influence ${\dot{V}}_{E}$, namely HR, f_B_, ACT, warm, age, sex, body mass index (BMI), FEV_1_/FVC, and height. We evaluated all models with one predictor, two predictors, and so on up to all nine predictors for a total of 511 possible models. For a given number of predictors, we selected the best model based on three criteria: the cross-validated percent error (described below), the Akaike Information Criterion (AIC), and the p-value of parameter estimates. Since the goal of this project was to develop a predictive model for estimating air pollution inhaled dose, special consideration was given to predictors that are widely available and easily measured in the field.

A five-fold cross-validation procedure was used to assess model performance. The 15 adolescent subjects were randomly divided into five groups such that each group was comprised of a training set of 12 subjects and a validation set of three. Parameter estimates were calculated based on the training sets, predictions were made for the validation sets, and then the predictions from all five validation sets were assembled and compared with observations. The cross-validated percent error was calculated as (predictions-observations)/observations.

## Results and Discussion

### Subject characteristics

Subject information is provided in Table 1. Participants were 15–18 years of age and in good health as might be expected of adolescents who engage in athletic training and sporting activities. Height, weight, and BMI were normal for this age range. Three subjects reported having received a physician’s diagnosis of asthma in their lifetime, but only one of these reported asthma symptoms in the previous year, and none were currently experiencing symptoms or taking asthma medication. Lung function measurements were compared to the standards established by the third National Health and Nutrition Examination Survey (NHANES III) [23] for the prediction of lung function values based on age, height, sex, and race. With the exception of two subjects (one of whom reported asthma symptoms in the previous year), FVC and FEV_1_ were well above the lower limit of normal values and were 85–115% of the NHANES III predicted values.

**Table 1:** Subject characteristics.

Age in years^a^	17.3(1.3)
Race/ethnicity^b^	
Non-Hispanic white	11(73)
African-American	1(6.7)
Hispanic	2(13)
Asian	1(6.7)
Sex^b^	
Male	9(60)
Female	6(40)
Height [cm]^a^	175(10)
Weight [kg]^a^	63(13)
BMI^a^	20.6(2.9)
Lung Function^c^	
FVC	4.34(0.78)
FEV_1_	3.80(0.61)
FEV_1_/FVC	0.88(0.051)
Lung Function^d^	
FVC	92(10)
FEV_1_	94(12)
FEV_1_/FVC	102(5.1)

^a^ Values are mean(SD)

^b^ Values are frequency(percentage)

^c^ Values are mean(SD) of the measured values (in L) or ratios

^d^ Values are mean(SD) of the percent of the NHANES III predicted values

### Selection of time-base for averaged values

As oxygen demand increases during physical activity, ${\dot{V}}_{E}$ is elevated by increasing both f_B_ and V_T_. In addition, airways may begin to dynamically compress during rapid exhalation, and this stimulates termination of exhalation and initiation of the following breath [24]. This phenomenon may manifest itself as a comparatively shallow breath followed rapidly by a larger breath. Given that ${\dot{V}}_{E}$ is calculated as the product of V_T_ and f_B_, on time scales of a few breaths or less, this may result in a noisy ${\dot{V}}_{E}$ signal. Longer averaging times will reduce this noise, but at the same time, true changes in HR and ${\dot{V}}_{E}$ occur on a time scale comparable to changes in activity level and oxygen demand. Therefore, choosing an averaging time that is too long may obscure intermediate data points and reduce statistical power. We evaluated selected models at all four averaging times and obtained similar parameter estimates. Estimates for a model containing HR and f_B_ as predictors are shown in Table 2. As can be seen from these results, predictive performance as assessed by cross-validation improves with longer averaging times while confidence intervals broaden due to reduced statistical power, although all estimates presented in Table 2 have p<0.0001. Given the similarity in model results across time-bases, we chose to focus on the 30-second average data set as a compromise between the extremes, and all the following results are from this data set.

**Table 2:** Influence of averaging time on parameter estimates. For parameter estimates, the first row is the estimate, and the second row is the 95% confidence interval. All have p<0.0001. Percent error refers to the difference between predictions and observations from cross validation, and values are mean(standard deviation).

	IB^a^	15 sec.	30 sec.	60 sec.
Intercept	-4.28	-4.20	-4.25	-4.34
	-5.40,-3.16	-5.35,-3.05	-5.49,-3.00	-5.73,-2.95
HR		0.057	0.060	0.063
	0.054,0.062	0.051,0.064	0.051,0.068	0.051,0.075
f_B_	0.24	0.24	0.23	0.21
	0.22,0.26	0.21,0.26	0.19,0.26	0.16,0.26
Percent error	12.3(54)%	8.9(31)%	8.1(29)%	7.2(26)%

^a^Individual Breath: HR, f_B_, and ${\dot{V}}_{E}$ averaged over the time interval since the previous breath (1–7 seconds).

### Model selection

Using ${\dot{V}}_{E}$/FVC as the dependent variable, we followed a brute force process for the selection of predictors by evaluating all 511 possible models containing from one to nine of the full set of predictors, including HR, f_B_, ACT, warm, age (years), sex (coded 0 for male and 1 for female), BMI, FEV_1_/FVC, and height (cm). For a given number of predictors, the best model was selected based on the cross-validated percent error, AIC values, and the p-value of parameter estimates, and the results for models using 30-second averaged data are shown in Table 3. For models containing between three and eight predictors, the model with the lowest percent error was not the same as the one with the lowest AIC. In this situation, we examined the p-value of the parameter estimates to determine if there was a higher degree of confidence in the estimates of a particular model based on the cumulative p-values. In all cases, we unambiguously identified one model as surpassing the other models for that number of predictors. By using all three of these criteria, the brute force selection process produced a pattern identical to both a forward and backward stepwise selection process; however, if only cross-validation percent error or AIC were independently used as model selection criteria, both stepwise selection processes would have produced predictor selection patterns different from each other as well as what is shown in Table 3.

**Table 3:** Results of general linear mixed models, displaying predictors included in each model, parameter estimates, p-values, model AIC values, and percent error. In all models, the dependent variable is ${\dot{V}}_{E}$/FVC. For parameter estimates, the first row is the estimate, and the second row is the p-value. Percent error refers to the difference between predictions and observations from cross validation, and values are mean(standard deviation).

	model 1	model 2	model 3	model 4	model 5	model 6	model 7	model 8	model 9
	-3.859	-4.247	-3.782	-2.85	11.65	15.9	12.0	14.30	15.44
	<0.0001	<0.0001	<0.0001	0.0019	0.0559	0.0908	0.0489	0.343	0.479
HR	0.101	0.0595	0.0531	0.0414	0.0420	0.0420	0.0418	0.0418	0.0418
	<0.0001	<0.0001	<0.0001	<0.0001	<0.0001	<0.0001	<0.0001	<0.0001	<0.0001
f_B_	-	0.226	0.229	0.227	0.229	0.228	0.227	0.227	0.227
		<0.0001	<0.0001	<0.0001	<0.0001	<0.0001	<0.0001	<0.0001	<0.0001
ACT	-	-	0.490	1.29	1.23	1.24	1.26	1.26	1.26
			0.0805	0.0037	0.0055	0.0053	0.0046	0.0046	0.0046
warm	-	-	-	0.566	0.540	0.544	0.554	0.554	0.554
				0.0200	0.0264	0.0253	0.0231	0.0231	0.0233
age	-	-	-	-	-0.845	-0.813	-1.17	-1.20	-1.19
					0.0094	0.0156	0.0022	0.0045	0.0087
sex	-	-	-	-	-	-0.0273	1.09	1.14	1.09
						0.518	0.202	0.223	0.369
BMI	-	-	-	-	-	-	0.241	0.243	0.241
							0.154	0.171	0.202
FEV_1_/FVC	-	-	-	-	-	-	-	-2.28	-2.69
								0.861	0.8546
height	-	-	-	-	-	-	-	-	-0.00481
									0.938
AIC	1244.0	1136.8	1134.4	1130.0	1124.6	1128.7	1122.3	1115.3	1119.1
Percent error	11.3(36)%	8.1(29)%	8.3(29)%	8.5(28)%	9.5(26)%	8.8(27)%	8.9(28)%	10.4(29)%	10.3(33)%

This discussion of the predictor selection process is essentially moot however since, as can be seen from the results in Table 3, addition of predictors beyond HR and f_B_ did not improve predictive performance. While the addition of ACT, warm, age, sex and BMI to the model lowered the AIC value, it actually worsened the cross-validated percent error. In all models containing both HR and f_B_, the p-value for these parameter estimates was less than 0.0001, and in the case of f_B_, the magnitude of the parameter estimate was practically unchanged by additional predictors. Since the dependent variable is ${\dot{V}}_{E}$ normalized by FVC, it was not surprising that factors with a strong influence on FVC (specifically height, sex, and age) would not be effective predictors of ${\dot{V}}_{E}$/FVC.

The rate of change of both f_B_ or V_T_ may be sensitive to whether activity intensity is increasing or decreasing [25]. We parameterized this effect by using the dichotomous indicator variable “warm”. In a 4-parameter model, the estimate for warm was 7, suggesting that for the same HR, f_B_ and ACT, ${\dot{V}}_{E}$/FVC would be a little more than half a unit higher in the cool-down period than in the warm-up period. Although this estimate was nominally significant with a p-value of 0.02, addition of this variable to the model slightly worsened cross-validated percent error compared to a 3-parameter model containing just HR, f_B_, and ACT. In a 3-parameter model with HR, f_B_, and warm, the estimate for warm is quite small (0.015) with a highly non-significant p-value of 0.92. For these reasons, we conclude that information concerning the direction of change of HR is not useful for predicting ${\dot{V}}_{E}$ in this population.

We additionally evaluated two other parameters that could plausibly influence ${\dot{V}}_{E}$/FVC: BMI and FEV_1_/FVC. Since it is well-known that fitness level improves lung function [26,27], we explored its effect on ${\dot{V}}_{E}$/FVC by using BMI as a proxy. Without exception, including BMI as a predictor worsened predictive performance. In a 3-parameter model including HR, f_B_, and BMI, the estimate for BMI was -0.064 (p = 0.71), leading us to the conclusion that BMI is not a useful predictor of ${\dot{V}}_{E}$/FVC in this population. A reduction in FEV_1_/FVC is associated with airway obstructions whereas airway restrictions tend to reduce both FEV_1_ and FVC[28]. Individuals with a low ratio could experience reduced V_T_ at high f_B_ compared to individuals with a normal or high ratio; however, our model results do not suggest an association between FEV_1_/FVC and ${\dot{V}}_{E}$/FVC. With respect to both BMI and FEV_1_/FVC, an obvious limitation of this study is that all subjects were healthy adolescent athletes with BMI and FEV_1_/FVC well within the normal range, and our results therefore do not preclude the possibility that ${\dot{V}}_{E}$/FVC may in other populations be influenced by fitness level or airway obstructive disease. As an alternative method for investigating the influence of FEV_1_, we evaluated all models using ${\dot{V}}_{E}$ normalized to FEV_1_ as the dependent variable rather than ${\dot{V}}_{E}$/FVC. The cross-validation results for these models were essentially the same as for ${\dot{V}}_{E}$/FVC, as might be expected since there was a very narrow range of FEV_1_/FVC ratios amongst our study subjects.

### Recommended model

**Fig 1:**
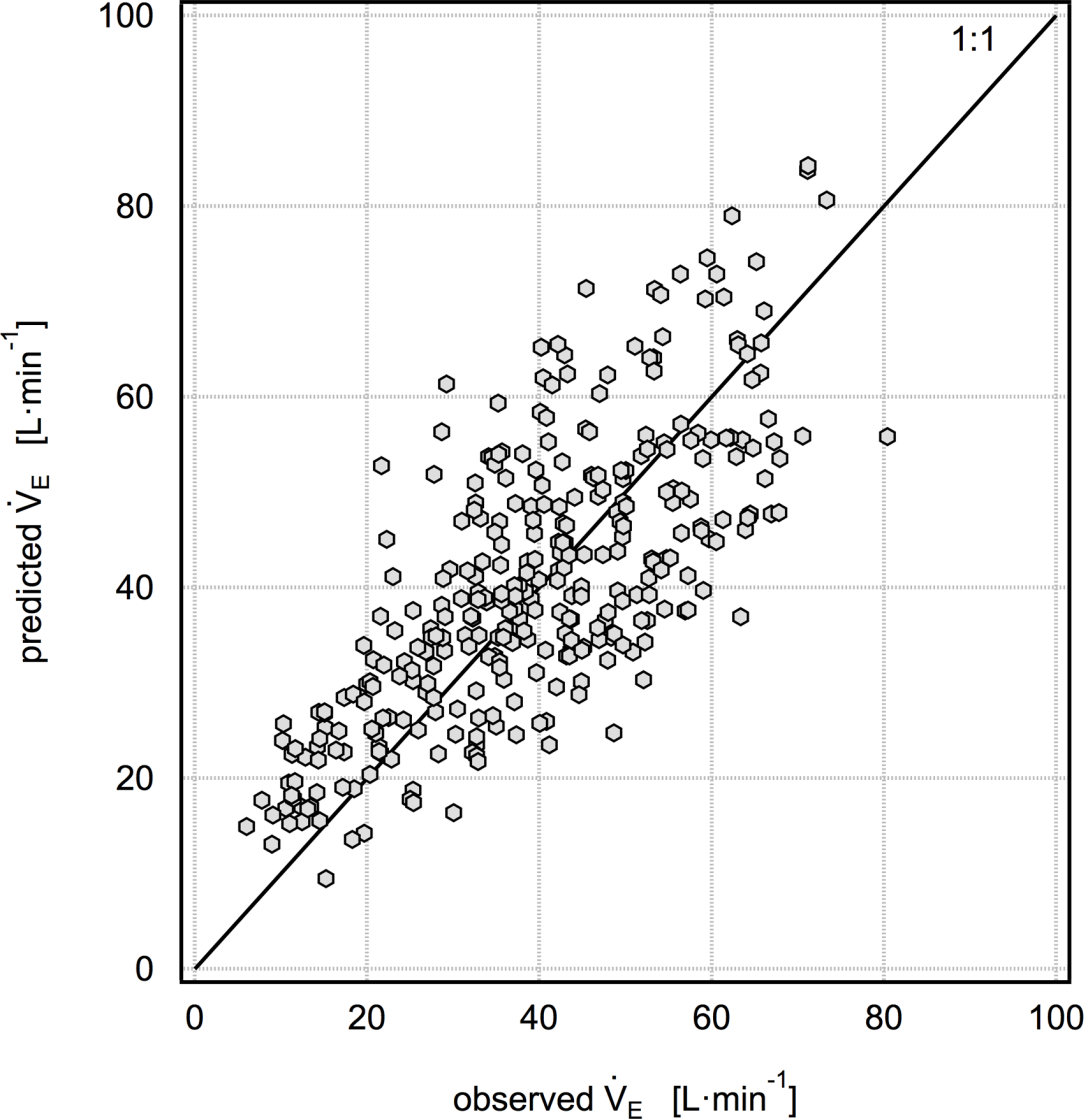
Five-fold cross validation results for the model ${\dot{V}}_{E}$/FVC = -3.859 + 0.101HR. The mean percent error for this model is 11.3±36%.

**Fig 2:**
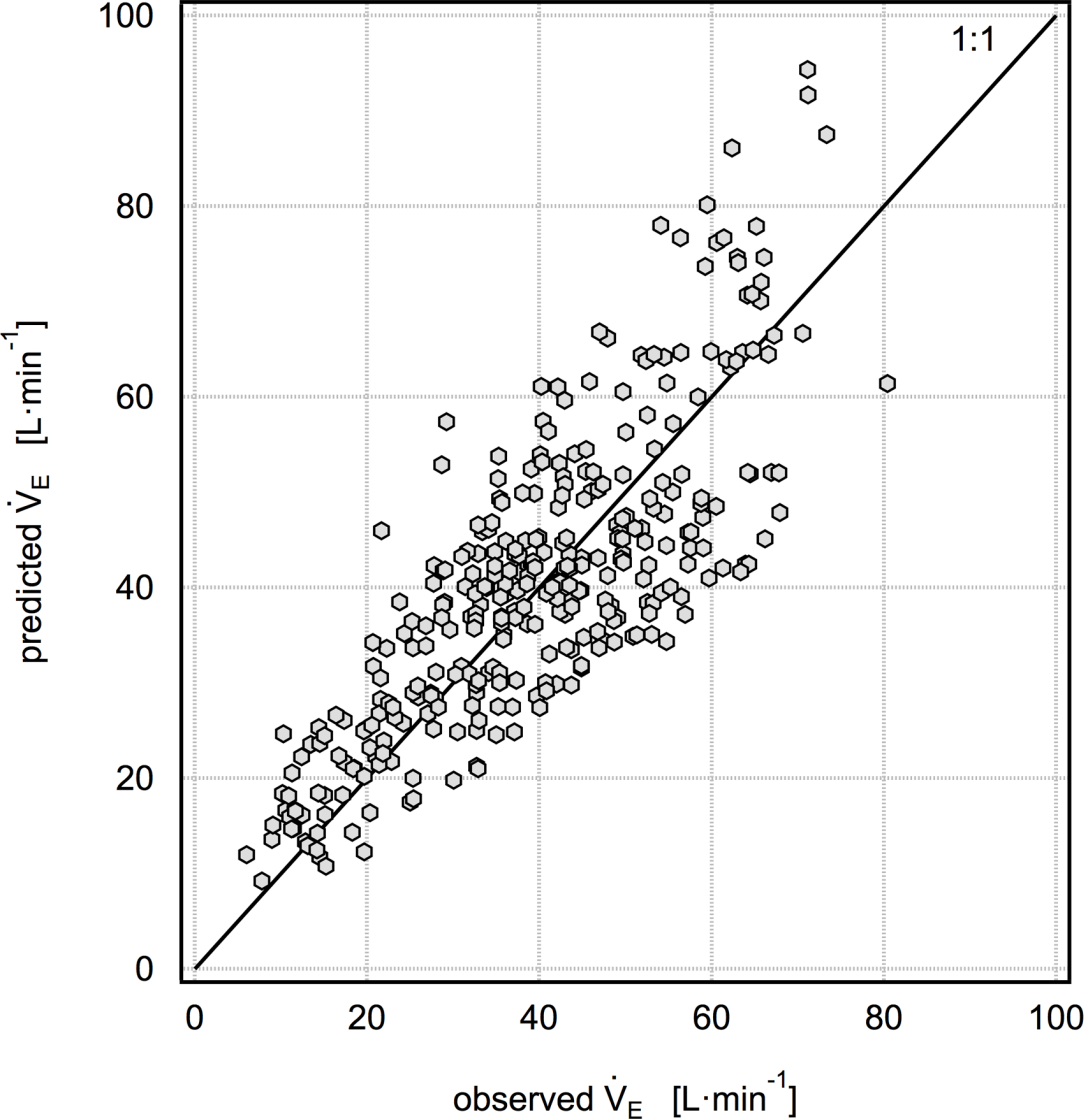
Five-fold cross validation results for the model ${\dot{V}}_{E}$/FVC = -4.247 + 0.0595HR + 0.226f_B_. The mean percent error for this model is 8.1±29%.

Given these considerations, our best or most practical predictive models are model 1: ${\dot{V}}_{E}$/FVC = -3.859+0.101HR (with a mean percent error of 11.3±36% as shown in Fig 1) and model 2: ${\dot{V}}_{E}$/FVC = -4.247+0.0595HR+0.226f_B_ (with a mean percent error of 8.1±29% as shown in Fig 2). Although the mean cross-validated percent error is higher for model 1 than model 2, it has the advantage of only using one predictor, HR, which is very easily measured in the field using a wide variety of inexpensive consumer devices. Model 2 additionally requires independent measurement of f_B_, which is easily obtained with unobtrusive devices in research studies but is unlikely to be available in studies without dedicated equipment. As HR increases from baseline to modest levels of physical activity, both V_T_ and f_B_ increase in an approximately linear fashion with respect to HR. In this scenario, it would seem that HR alone would be sufficient to predict ${\dot{V}}_{E}$/FVC. As HR continues to increase during more vigorous activity, V_T_ plateaus and ${\dot{V}}_{E}$ may only be increased by increasing f_B_ [24]. We examined the possibility that a one-predictor model with HR would have a higher predictive performance at HR<120, 130 or 140; however, we did not find this to be the case. We also examined the possibility that a one-predictor model with f_B_ would be advantageous, but we did not find this to be true. The results for this model are ${\dot{V}}_{E}$/FVC = -1.913+0.439f_B_ (with a mean percent error of 9.7±38%). While this is marginally better than model 1, it is not better than model 2, and it is unlikely that there will be a situation in a field campaign where f_B_ data is available while HR data is not.

**Fig 3:**
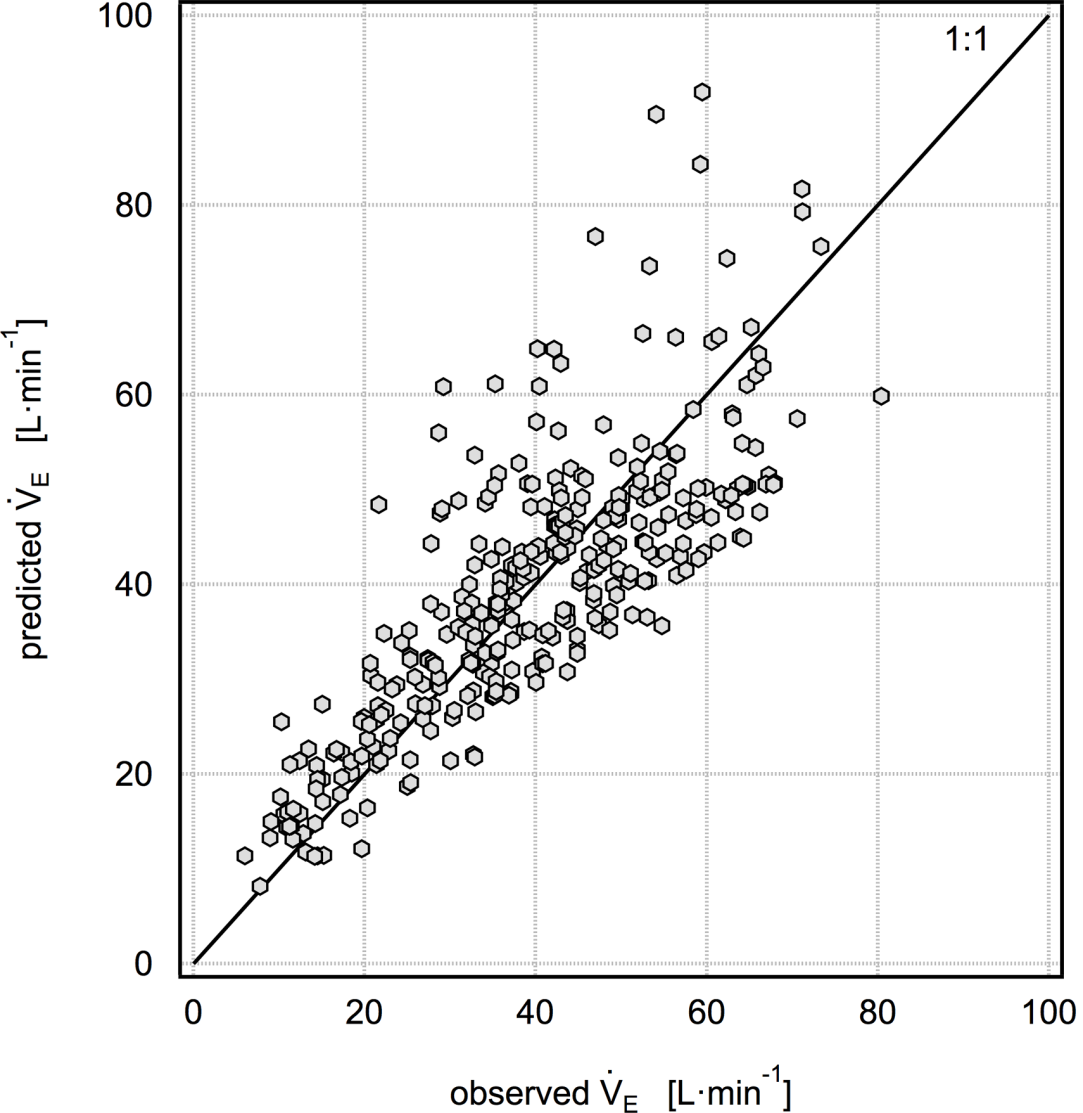
Five-fold cross validation results for the model ${\dot{V}}_{E}$/(predicted FVC) = -4.148 + 0.0535HR + 0.221f_B_, where predicted FVC is calculated using NHANES III coefficients. The mean percent error for this model is 6.0±28%.

Both models 1 and 2 require measurements of FVC in order to calculate ${\dot{V}}_{E}$ and inhaled dose; however, in the absence of spirometry, reasonable estimates of FVC may be calculated for healthy individuals using the NHANES III [23] coefficients and subject height, age, sex, and race information. Fig 3 shows the equivalent model 2 normalized to NHANES III predicted FVC. Although the mean cross-validated percent error is lower in this model than for model 2 normalized to measured FVC, this result is dependent on the degree to which measured FVC matched predicted FVC in this study population.

### Use for estimation of inhaled dose

**Fig 4:**
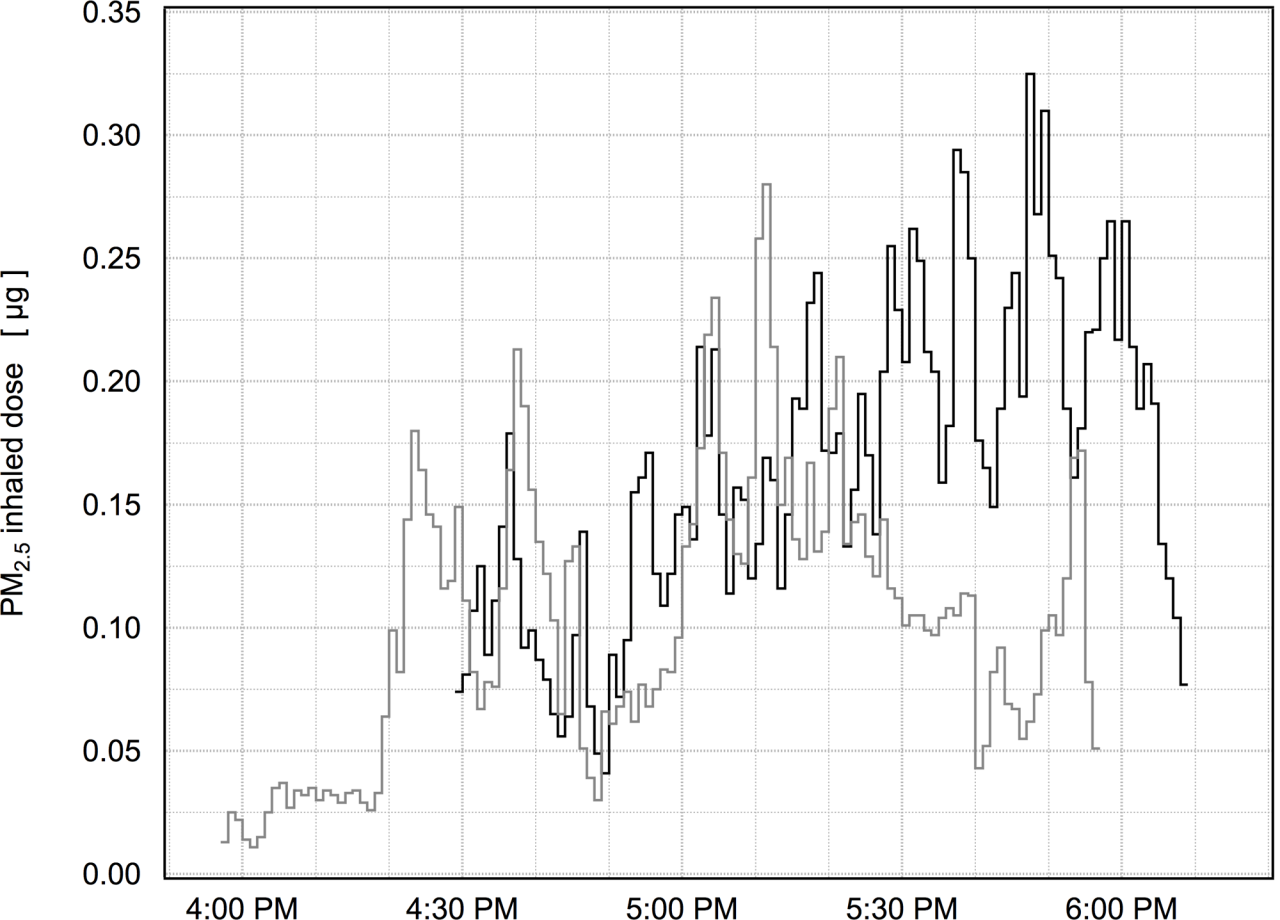
Time-resolved inhaled does of PM_2.5_ for a sprinter (black line) and a soccer player (gray line) during after-school practice. The cumulative inhaled dose was 16.6 μg for the sprinter, and 12.3 μg for the soccer player.

As a demonstration of the practical use of this model for estimating air pollution inhaled dose, we applied model 2 to data collected as part of the parent study of air pollution and physical activity involving high school sports teams. We selected at random a sample day on which the soccer team was conducting a practice on the school field while the track and field team practiced on the encircling track. Participants of both teams were simultaneously present at this location, and air pollution measurements were performed with 1-minute time resolution, including the mass of particular matter less than 2.5 μm (PM_2.5_). Fig 4 shows the time series of PM_2.5_ inhaled dose for a soccer player and a sprinter on the track team. Both subjects were males of the same age with approximately the same height and FVC. Some minute-to-minute changes in inhaled dose were related to changes in the PM_2.5_ concentration, but most were related to changes in ${\dot{V}}_{E}$ in response to physical activity. The exposure periods were overlapping, and the mean PM_2.5_ concentration was 3.9 μg·m^-3^ during the sprinter’s 105-minute exposure period and 3.4 μg·m^-3^ during the soccer player’s 90-minute exposure period. The corresponding exposures (concentration·time) were 413 and 420 μg·min·m^-3^ respectively. Although the exposures were nearly the same, the sprinter inhaled 4.3 m^3^ of air while the soccer player inhaled 3.4 m^3^, and the corresponding inhaled doses were 16.6 μg (0.19 μg/kg body mass) and 12.3 μg (0.22 μg/kg body mass) respectively. These subjects were very active during this period with both HR and f_B_ at times approaching their maximum values; however, if the subjects had remained at rest during the exposure period with baseline ${\dot{V}}_{E}$, their inhaled dose of PM_2.5_ would have been 5.0 and 4.8 μg respectively, or about a third of the dose during activity. There were only slight differences in dose estimates using model 1 (with HR as the only predictor). The model 1 inhaled dose estimate was found to be 0.6% lower for the sprinter and 2.4% higher for the soccer player.

### Limitations

This study involved a panel of healthy adolescents, and therefore our results may not be generalizable at the population level. Although age was not found to be an important predictor of ${\dot{V}}_{E}$/FVC, this may be due to the narrow age range of study participants, none of whom had yet reached adulthood. Given that FVC steadily declines with age once adulthood is reached, it is possible that ${\dot{V}}_{E}$/FVC may also be affected by age in adults. Similarly, these results may not be applicable to pre-adolescent children. Although three subjects reported a history of asthma, none were currently experiencing symptoms, and no subjects had obstructive airway disease. In an exploratory analysis, we excluded the three subjects with a history of asthma, and the result was not significantly different than excluding at random three subjects without a history of asthma. Consequently, our results do not offer any insight into the role of airway disease on ${\dot{V}}_{E}$/FVC.

## Conclusion

We describe a novel method to estimate ${\dot{V}}_{E}$ for healthy adolescents using only measurements of HR and f_B_. Since individuals of various sizes and fitness levels have widely varying lung capacities, the key feature of this method is to normalize ${\dot{V}}_{E}$ by FVC. It is preferable to have accurate and recent spirometric measurements of FVC, but in their absence, we found little difference in model predictions when using FVC predicted using NHANES III coefficients for healthy subjects with normal lung function. A five-fold cross-validation procedure found a mean percent error of 8.1% for a two-predictor model including HR and f_B_ and 11.3% for a model using HR as the sole predictor. Although additional work is needed to refine these estimates and examine these associations in other populations, the method described here is a substantial improvement over existing methods for estimating ${\dot{V}}_{E}$ in natural settings. When coupled with time-resolved air pollution measurements, this method allows estimation of the inhaled dose of air pollution and may be particularly useful in estimating changes in inhaled dose resulting from physical activity.

## Supporting Information

S1 File The dataset of 30-second averaged data is included in an online supplement. (ZIP)
